# Fast Adsorption of Short and Long-Chain Per- and Polyfluoroalkyl Substances from Water by Chemically Modified Sawdust

**DOI:** 10.1021/acsestwater.5c00960

**Published:** 2026-01-28

**Authors:** Behnia Bitaraf, Md. Nahid Pervez, Tao Jiang, Marina Maria Ioanniti, Haralabos Efstathiadis, Mehmet V. Yigit, Yanna Liang

**Affiliations:** a Department of Environmental and Sustainable Engineering, University at Albany, State University of New York, Albany, New York 12222, United States; b Department of Nanoscale Science and Engineering, University at Albany, State University of New York, Albany, New York 12222, United States; c Department of Chemistry, University at Albany, State University of New York, Albany, New York 12222, United States

**Keywords:** multicomponent PFAS mixture, isotherm, *m*-phenylenediamine polymerization, regeneration, reusability, river water

## Abstract

To remove per- and polyfluoroalkyl substances (PFAS) from water, this study focused on synthesizing a sawdust-based adsorbent through KMnO_4_ oxidation and coating m-phenylenediamine (mPD) onto the sawdust’s surface. The resulting sawdust/MnO_2_/PmPD was able to remove >90% of nine target PFAS and >80% of GenX spiked at 10 ppb in deionized water. When added to river water samples, the capture of long-chain PFAS remained basically the same. This was in line with the observations that environmental factors, such as a change of pH between 4.0 and 11.0, the presence of natural organic matter in the range of 0 and 100 mg L^–1^, and the presence of bicarbonate, nitrate, and chloride, each at 1 mM, did not affect the removal of long-chain PFAS significantly. The low-cost nature of this sorbent was further strengthened by its regenerability and reusability for at least five cycles. To improve the sorption performance, especially for short-chain PFAS, further modification of the sawdust/MnO_2_/PmPD will need to be performed based on the revealed mechanisms underlying PFAS capture. Overall, at this stage, the sawdust/MnO_2_/PmPD material is ready to be used for removing PFAS from surface water.

## Graphical abstract

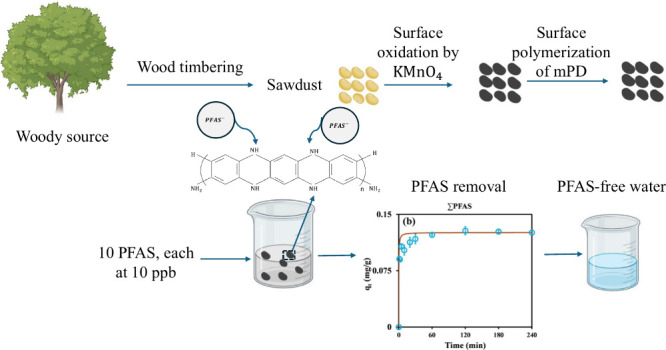

## 1 Introduction

Per- and polyfluoroalkyl substances (PFAS) are a class of synthetic compounds that include fluorinated carbon chains.^1^ The most often used PFAS are perfluoroalkyl carboxylic acids (PFCAs) and perfluoroalkyl sulfonic acids (PFSAs), which together are known as perfluoroalkyl acids (PFAAs). PFAAs are amphiphiles that have a polar acid headgroup and a hydrophobic perfluorinated carbon tail.^2^ Due to their strong chemical and mechanical stability, PFAS have been extensively used in commercial products, leading to their presence in water bodies, soil, living species, and eventually affecting the environment and human health.^3,4^

Finding appropriate and cost-effective ways to remediate PFAS is thus of the utmost importance. In this context, adsorption has been identified as one of the feasible options for capturing PFAS from aqueous media. Currently, numerous materials have been researched as adsorbents for PFAS removal, of which activated carbon (AC), carbon nanotubes (CNT), biochar, and clay have been investigated intensively.^5–7^ Among carbon-based adsorbents, granular AC (GAC) has been used on household and commercial scales for PFAS removal from drinking water. GAC, in comparison with newly developed adsorbents, is relatively inexpensive,^8^ with some being regenerable.^9^ However, GAC suffers from an insufficiency in capturing short-chain PFAS^10^ and also has slow adsorption kinetics.^11^ Additionally, the production of GAC requires a temperature of up to 400–1200 °C and the use of strong acids or bases that are harmful to the environment.^12^

As an alternative to the aforementioned adsorbents, sawdust has garnered much attention in removing PFAS from water bodies.^13^ Sawdust is a byproduct of the furniture industry,^14^ the operation of milling wood,^15^ and timbering. Sawdust typically contains cellulose, hemicellulose, and lignin. Sawdust, in essence, due to the lack of functional groups, is not a good material for PFAS removal by itself. Its abundance, environmentally friendly nature, and cost-effectiveness,^16^ however, make sawdust-derived materials an attractive choice as an adsorbent for PFAS remediation. For instance, Niaz et al. used pine wood biochar coated with MgFe_2_O_4_ nanoparticles to remove PFOS and PFOA from aqueous environments. However, the toxicity and the potential hazard of the magnetic nanoparticles (MNPs) generated during the synthesis process are among the most significant problems and could endanger both the environment and human health.^17^ In another study, Yang and Cannon^18^ utilized pine sawdust to obtain activated carbon for PFOA removal. They used a hydrothermal process, followed by pyrolysis at 900 °C. Aside from the high energy consumption of the fabrication process, this study targeted PFOA only. Yu^19^ used sawdust to produce biochar coated with polypyrrole. While they studied a multicomponent PFAS solution, the investigated PFAS concentration range of 1–200 mg L^–1^ is unlikely to be found in our environment. PFAS as surfactants can form micelles once reaching 0.001–0.01 of their critical micelle concentration (CMC).^20^ For PFOS and PFOA, the CMCs are 8 and 25 mM, respectively.^21^ Thus, insight gained from ref 19 with PFAS in mg/L concentrations cannot be directly applied to applications where PFAS are at μg/L or ng/L concentrations.^22^

In addition to producing biochar as an adsorbent from sawdust, another approach for sawdust modification is to provide functional groups on its surface. This can be accomplished through polymerizing conductive polymers, such as polyaniline, polypyrrole, and poly­(*m*-phenylenediamine) (PmPD).^23^ This polymerization is enabled by the presence of active sites on the sawdust surface.^24^

Given that PFAS share a common C–F backbone, have low p*K*_a_ values, and are negatively charged at neutral pH, we hypothesized that an adsorbent with a hydrophobic structure and positively charged functional groups would be able to capture PFAS effectively through both hydrophobic and electrostatic interactions. To prove this hypothesis, we chose to polymerize mPD on sawdust. This considers the hydrophobic nature of sawdust, the benzene rings, and the presence of NH_2_ in the structure of conductive polymers, such as PmPD.^25^

To synthesize this new material, as detailed below, KMnO_4_ was first used to oxidize the surface of sawdust. Upon oxidation of sawdust, KMnO_4_ was reduced to MnO_2_ particles and deposited on the surface of the sawdust. MnO_2_ then acted as an oxidant to initiate the polymerization of mPD monomers directly on the sawdust interface since the oxidizing reagent (MnO_2_) was localized on the sawdust surface. Other oxidants, such as ammonium persulfate (APS), unlike KMnO_4_, do not leave behind an oxidizing layer (i.e., MnO_2_ particles) on the sawdust surface; therefore, the surface polymerization of mPD does not occur.

In this study, we evaluated the effectiveness of PmPD-modified sawdust without any pyrolysis step in removing a mixture of 10 PFAS at environmentally relevant concentrations. To understand the sorption mechanisms, the sawdust/MnO_2_/PmPD sorbent was characterized in detail using scanning electron microscopy and energy-dispersive X-ray spectroscopy (SEM/EDX), Fourier-transform infrared spectroscopy (FT-IR), X-ray photoelectron spectroscopy (XPS), zeta point charge (ZPC), and X-ray diffraction analysis (XRD) techniques. Furthermore, the potential influence of environmental factors such as pH, coexisting ions, and organic matter represented by humic acid (HA) was elucidated. The use of sawdust/MnO_2_/PmPD for removing PFAS in river water was performed, as well. Finally, based on all fundamental understanding and insight learned from the study on the sorbent’s regenerability and reusability, the mechanisms underlying PFAS sorption by the sawdust/MnO_2_/PmPD were proposed. To the best of our knowledge, this is the first study adopting chemically modified sawdust for capturing PFAS in surface water.

## 2 Experimental/Methods

### 2.1 Materials

The list of materials and characterizations of PFAS used in this study is summarized in Tables S1–S3 in the Supporting Information.

#### 2.2.1 Synthesis of the Sawdust/MnO_2_ Composite

Sawdust, derived from hardwood and purchased from Shannon’s sawmill, was sieved through 1, 0.84, 0.42, and 0.15 mm openings. The majority of particles were between 0.84 and 0.42 mm, and this fraction was used for preparing the adsorbent. To begin with, 0.5 mL of H_2_SO_4_ was mixed with 50 mL of DI water, and then 0.5 g of KMnO_4_ was added to the mixture. After dispersal of KMnO_4_ particles, 0.5 g of the raw sawdust was introduced to the solution. The resulting solution was kept on a magnetic hot plate for 15 min at 100 °C. In this step, KMnO_4_ oxidized the surface of sawdust, leading to its coverage by MnO_2_ particles. The MnO_2_ then served as an oxidizing agent for polymerizing the mPD in the next step. This composite was referred to as sawdust@MnO_2_.

#### 2.2.2 Synthesis of the Sawdust/MnO_2_/PmPD

To polymerize the mPD on the surface of sawdust, a solution containing 20 mL of DI water, 0.5 mL of HCl, and 0.3 g of mPD particles was prepared. Following the dissolution of the mPD particles, the sawdust@MnO_2_ was added to the solution and kept on the magnetic stirrer for 1 h to complete the polymerization step. In this step, the MnO_2_ particles coated on the surface of the sawdust acted as an oxidant to initiate the polymerization of the mPD monomers on the surface of the sawdust/MnO_2_.

### 2.3 Characterization Methods

In order to inspect the potential functional groups on the adsorbent, FT-IR analysis (PerkinElmer Spectrum 100, Waltham, MA, USA) was conducted in the range of 4000 to 650 cm^–1^. To investigate the crystallinity of the adsorbent, an XRD technique (Rigaku MiniFlex 6 G, Rigaku Corporation, Tokyo, Japan) was performed. XPS (PHI Quantera II, MN, USA) was applied to investigate the configuration of bonds on the adsorbent’s surface. To illustrate the morphology and structure of the adsorbent, an SEM analysis (Zeiss LEO 1550, Oberkochen, Germany) was employed. Moreover, to understand the surface elemental composition of the adsorbent, an EDX characterization (Bruker Quantax XFlash 6, Billerica, MA, USA) was adopted. Finally, to investigate the net surface charge of the material, zero point charge analysis (pH_ZPC_, Malvern Panalytical Ltd., Malvern, UK) in the pH range of 2–12 was executed.

### 2.4 Adsorption Experiments

Adsorption experiments were conducted in 50 mL polypropylene centrifuge tubes (Corning Inc., Corning, NY, USA). Briefly, a mixture of ten PFAS (PFBS, GenX, PFHxA, PFHxS, PFHpA, 6:2 FTSA, PFOS, PFOA, PFNA, and PFDA) was added to 50 mL of DI water. The concentration of each PFAS was 10 μg L^–1^. Before introducing an adsorbent (e.g., sawdust, sawdust/MnO_2_, and sawdust/MnO_2_/PmPD), 500 μL of the PFAS solution was collected from each tube. These samples were used to measure the true PFAS concentrations at the beginning of the adsorption. After adding 0.05 g of a target adsorbent to the solution, all tubes were loaded onto a rotary shaker set at 120 rpm for 3 h. Each sorbent was tested with at least two replicates. Subsamples collected at different time points were centrifuged for solid–liquid separation. The supernatant after passing 0.2 μm nylon filters was analyzed by an Agilent Technologies 1290 Infinity II LC system paired with a 6470 Triple Quad Mass Spectrometer (LC-MS/MS, Santa Clara, CA, USA) following our reported procedures.^26^ The details are provided in Text S1 **.**

### 2.5 Sorption Kinetics

Kinetic experiments were conducted within 4 h, at a pH of 5.7 with a mixture of ten PFAS, each at 10 μg L^–1^ in the presence of 1 g L^–1^ adsorbent. In order to evaluate the rate of PFAS adsorption to the adsorbent, three different kinetic models were applied as follows: eqs 1–3:

$$q_{t}=q_{e}(1-e^{-k_{1}t)}$$ 1

$$q_{t}=k_{2}q_{e}^{2}t/\left(1+k_{2}q_{e}t\right)$$ 2

$$q_{t}=k_{d}t^{0.5}+C$$ 3

where *q_t_* is the adsorption capacity at a given time, *q_e_* stands for the adsorption capacity at the equilibrium, *k*_1_, *k*_2_, and *k*_3_ represent kinetic coefficients of pseudo-first-order (PFO), semi-second-order (PSO), and interparticle diffusion models, respectively, and *t* is time.

### 2.6 Sorption Isotherm

Isotherms of adsorption were evaluated by using mixtures of PFAS solutions with PFAS concentrations ranging between 10 and 200 μg L^–1^. Typical isotherms, Langmuir, Freundlich, Sips, and Toth, were applied to model the experimental data. The equations are as follows: eqs 4–7 **:**

$$q_{e}=K_{L}q_{m}C_{e}/\left(1+K_{L}C_{e}\right)$$ 4

$$q_{e}=K_{F}C_{e}^{1/m}$$ 5

$$q_{e}=q_{m}{\left(K_{s}C_{s}\right)}^{1/n}/\left[1+{\left(K_{s}C_{e}\right)}^{1/n}\right]$$ 6

$$q_{e}=q_{m}K_{T}C_{e}/{\left[1+{\left(K_{T}C_{e}\right)}^{t}\right]}^{1/t}$$ 7

Above which, *q*_*e*_ is the adsorption capacity at equilibrium (mg g^–1^), *q*_*m*_ is the maximum adsorption capacity (mg g^–1^), *K_L_* represents the Langmuir constant associated with adsorption capacity (L/μg), and *K_F_* is the Freundlich constant attributed to the adsorption capacity and energy of the adsorption. Regarding the Sips and Toth models, *K*_*s*_ (L/μg) and *K*_*T*_ (L/μg) are constants related to adsorption affinity. Finally, *m* and *n* show the favorability and heterogeneity of the system, respectively.^26^

### 2.7 Test on Regeneration and Reuse

The sawdust/MnO_2_/PmPD was regenerated in three rounds, each 30 min, using 1% methanolic ammonium hydroxide (50% Methanol).^27^ First, the spent adsorbent was mixed with 10 mL of the regeneration agent and shaken on a rotary shaker. After that, the rinsed sorbent was collected after centrifugation. The second and third rounds were performed in the same way as the first round. The supernatant from each round was measured for the 10 PFAS, and the resulting concentrations were used to calculate the mass of PFAS desorbed by the methanol rinse. The desorbed adsorbent upon drying was used in the second cycle for capturing the same set of PFAS. Following a methanol rinse for PFAS desorption, the resulting material was dipped in the spent mPD solution derived from the initial synthesis of the sawdust/MnO_2_/PmPD, dried, and used for the next cycle of PFAS removal. The dipping step was referred to as the remodification of the spent sorbent. A total of five cycles was performed to verify the reusability of the sawdust/MnO_2_/PmPD.

### 2.8 Removal of PFAS in Non-DI Water Matrices

To understand the effect of pH, natural organic matter (NOM), and ionic strength on the removal of PFAS in water, a range of pH between 4.0 and 11.0, humic acid spiking at 0–100 mg/L, and spiking of sodium carbonate, sodium nitrate, and sodium chloride, each at 1 mM individually, was studied. Each PFAS was targeted at 10 μg L^–1^. To evaluate the removal of PFAS in real water, river water was collected from the Hudson River, NY, and characterized in detail, as shown in Table S4. After it was spiked with a mixture of PFAS, with each spike at 10 μg L^–1^, sorption tests similar to those described above were conducted.

## 3 Results and Discussion

### 3.1 Sorption of PFAS by Sawdust and Sawdust/MnO_2_

Sawdust by itself did not capture any target PFAS. After MnO_2_ modification, there was a trivial increase in the removal of PFBS, 6:2 FTSA, PFOS, PFNA, and PFDA (Figure S1). This limited removal signified the need for further modification using PmPD. The following sections focus on sawdust/MnO_2_/PmPD only.

### 3.2 Kinetic Studies of PFAS Removal by the Sawdust/MnO_2_/PmPD

Kinetic studies were performed within 4 h, with the initial concentration of 10 μg L^–1^ for each PFAS, and an adsorbent dose of 1 g L^–1^. As can be seen in Figure 1a, using PFOA as a representative, the majority of adsorption was completed within the first 30 min, and the adsorbent reached the equilibrium state at 1 h. This finding shows a faster kinetics of the sawdust/MnO_2_/PmPD over conventional GAC sorbent, which typically necessitates 4–240 h for reaching equilibrium.^28^ The same phenomenon was observed for the total PFAS (Figure 1b). These observations hinted that the first stage of rapid sorption was due to the presence of abundant active sites on the surface of the adsorbent.^29^ As the process continued, these sites were saturated by PFAS, and the kinetics slowed and finally reached the equilibrium state. According to the fitted kinetic models, the PSO showed better fitting over PFO and interparticle diffusion, and the *R* ^2^ values ranged from 0.97 to 1.0. All related information and linear fittings are available in Figures S2–S4.

**Figure 1:**
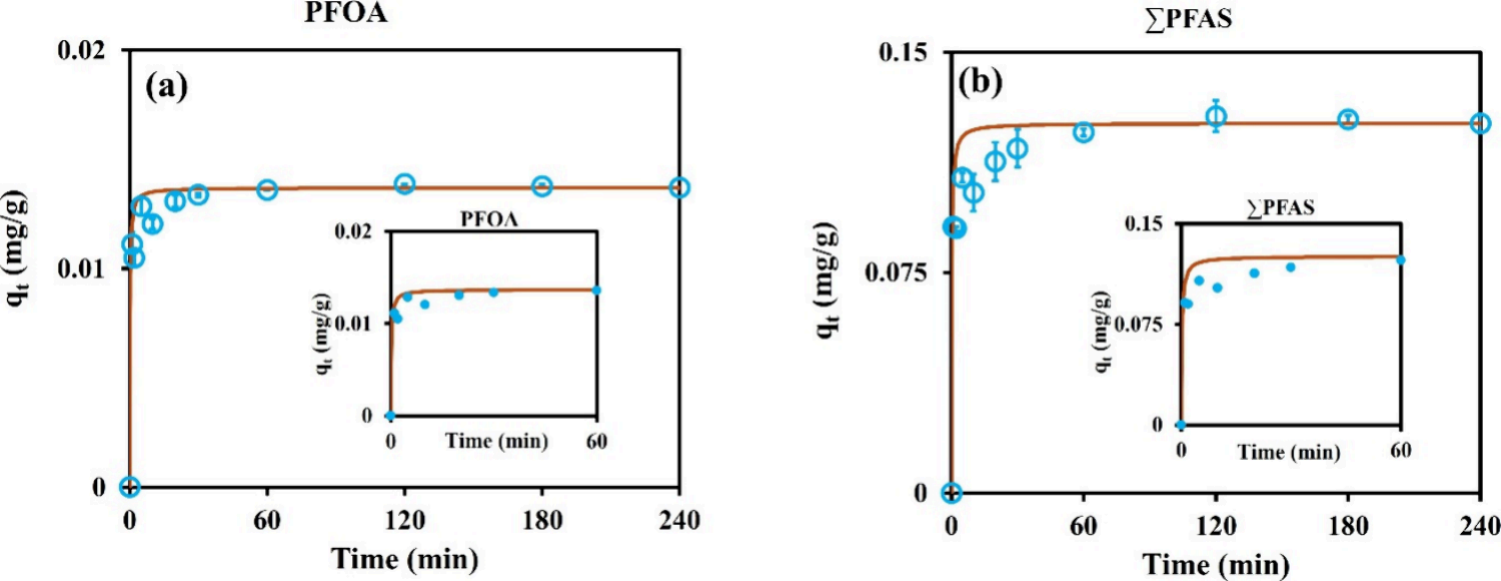
Sawdust/MnO_2_/PmPD kinetic study of (a) PFOA as a representative and (b) total PFAS.

### 3.3 Isotherm Modeling

In order to investigate the adsorption isotherm, four models were applied, as shown in Figure 2. The Langmuir model assumes a monolayer adsorption of the adsorbate and adsorbent, and no interactions between adsorbed molecules.^30^ The Freundlich model assumes multilayer interactions between an adsorbent and adsorbate molecules taking place on heterogeneous surface sites.^31^ Whereas, the Sips and Toth isotherm models integrate both Langmuir and Freundlich models and cover monolayer sorption at high concentrations and behave similarly to the Freundlich model when the adsorbate’s concentration is low.^11,32^ For this study, the *C_e_* and *q_e_* values of all PFAS (ΣPFAS) were calculated, as it was assumed that these PFAS shared similar physicochemical properties. As indicated in Tables S5 and S6, at higher concentrations, the predicted values from the Langmuir model matched the experimental data more closely than the Freundlich model. Meanwhile, the Sips isotherm performed better than the others and had an *R* ^2^ = 0.98. Therefore, it can be hypothesized that sawdust/MnO_2_/PmPD has a heterogeneous layer, and PFAS molecules can bind to the surface with different affinities through different mechanisms. A detailed discussion regarding the possible mechanisms is presented in Section 4 below.

**Figure 2:**
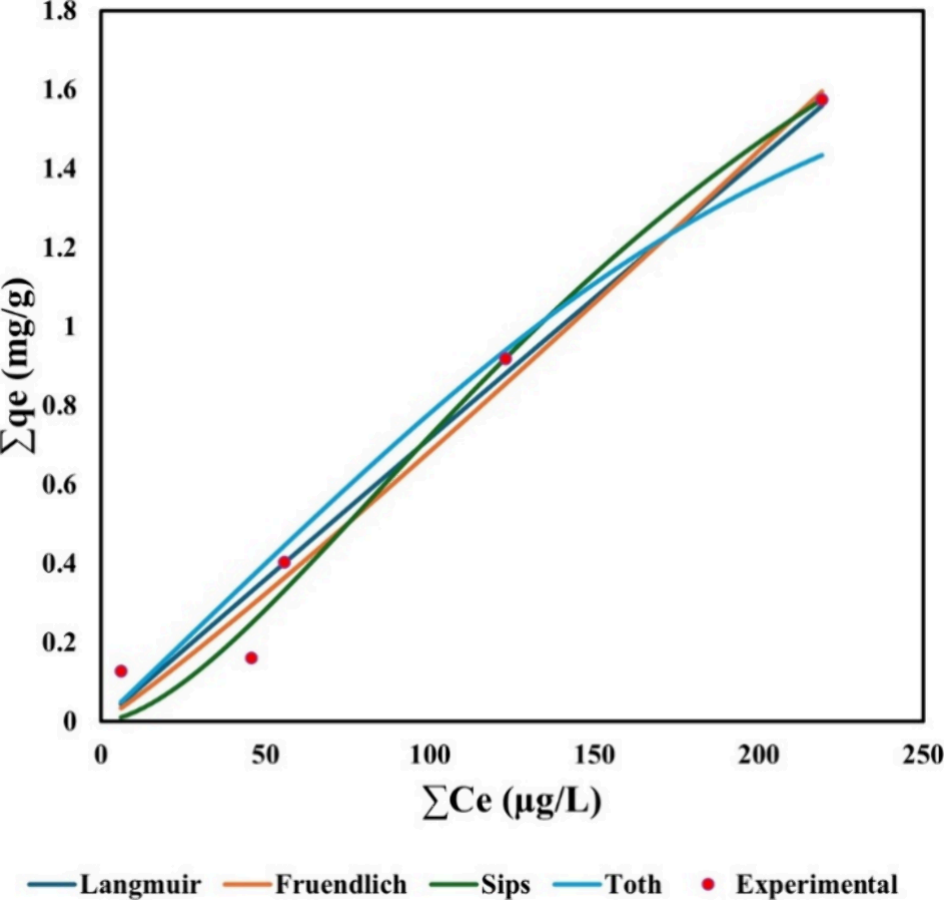
Isotherm studies of the sawdust/MnO_2_/PmPD.

### 3.4 Characterization of the Sawdust/MnO_2_/PmPD before and after Adsorption

FT-IR analysis was performed to detect the presence of chemical functional groups on the structure of the adsorbent. According to Figure 3a, bands typically appearing around 600 cm^–1^ correspond to MnO_2_ particles, and the broad band of 3370 cm^–1^ is attributed to the NH_2_ groups of PmPD, proving the successful surface oxidation by MnO_2_ and surface polymerization of mPD monomers on sawdust. FT-IR analysis of the postadsorption samples revealed the peaks of −CF_2_ and −CF_3_, which provided evidence of the adsorption of PFAS on the surface of the sawdust/MnO_2_/PmPD (Figure 3b).

**Figure 3:**
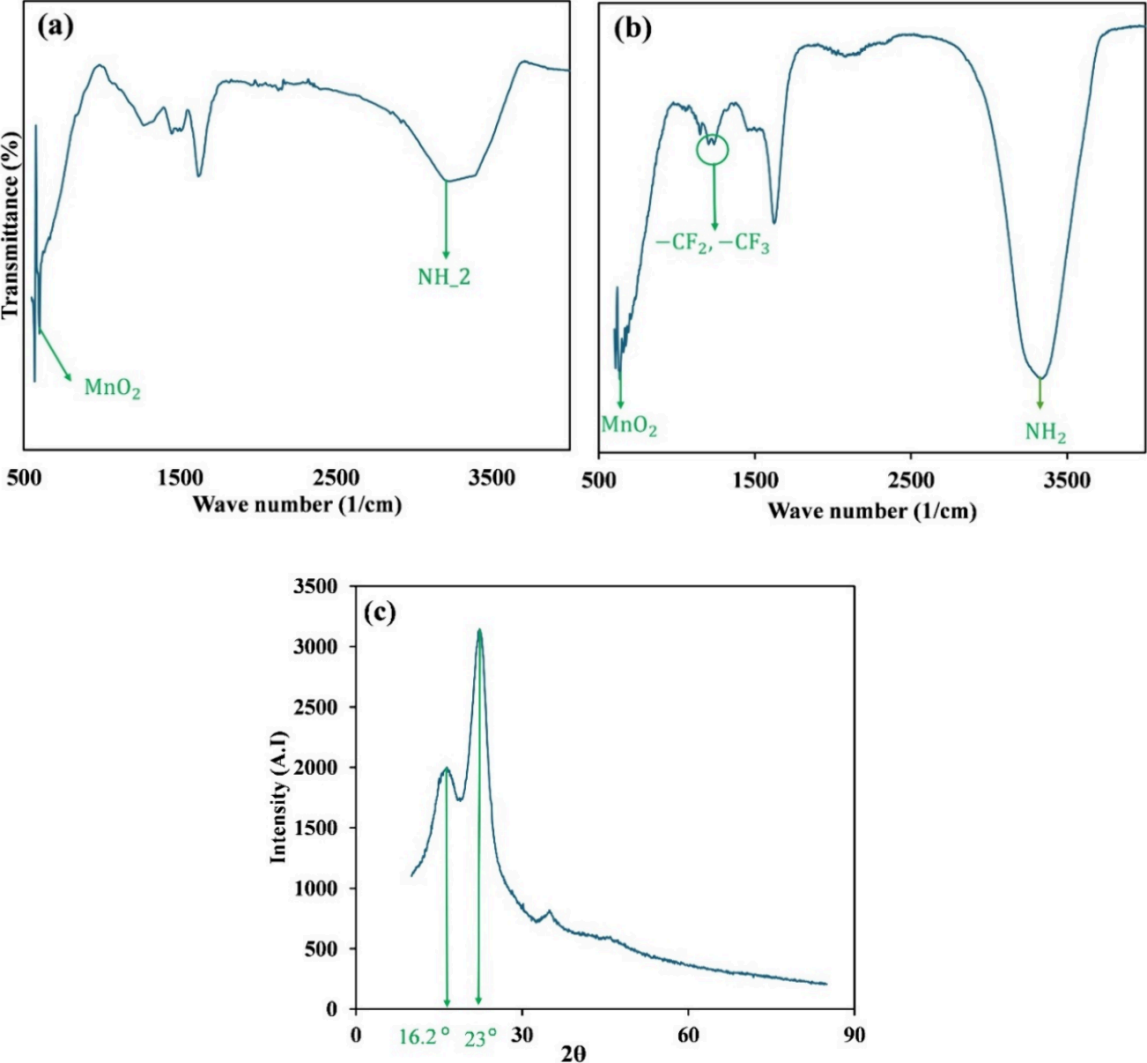
FT-IR analysis of sawdust/MnO_2_/PmPD, before adsorption (a), after adsorption (b), and XRD pattern of sawdust/MnO_2_/PmPD (c).

According to the XRD pattern, Figure 3c, of the synthesized adsorbent, the peak around 2θ = 16.2° is related to the cellulose structure of the sawdust.^33^ Moreover, the observed peak around 23° is typically attributed to the amorphous structure of PmPD.^34^

The XPS analysis (Figure S5) was conducted to determine the bonds and chemical groups constituting the configuration of the sawdust/MnO_2_/PmPD adsorbent at different stages. As depicted in Figure S6, the spectra of C 1s revealed three different peaks at 285, 286.025, and 288.052 eV, corresponding to benzoic rings of mPD, which are linked to the nitrogen (C–N) and C=O bonds.^35^ For O 1s XPS, the peak around 533.39 eV was likely attributed to adsorbed water, and the peak at 531.53 eV was indicative of the C–O groups of sawdust.^36^ Additionally, the presence of N was detected by XPS analysis (Figure 4 and Figure S6). In this regard, N 1s was resolved into three distinct peaks at 399.91, 401.168, and 400.202 eV, which were assigned to NH, O=C–NH, and NH_3_^+^, respectively.^37,38^ After exposure to PFAS, several notable changes were observed. The C 1s spectrum showed the emergence of a new peak at 292.2 eV, characteristic of CF_*x*_ (C–F2, C–F3) species, providing direct evidence of adsorbed perfluoroalkyl groups. Correspondingly, the F 1s spectrum displayed a strong peak at 689.58 eV, confirming the presence of organic fluorine. The N 1s peaks shifted slightly to 400.2 and 402.1 eV, consistent with electrostatic interactions or ion pairing between protonated amines and PFAS anions. The O 1s spectrum of 533.4 eV remained essentially unchanged, indicating minimal contribution of Mn-oxygenated functional groups to adsorption. Together, the unique appearance of strong F 1s and CF_*x*_ features, and the shifts in the N 1s region indicate that PFAS molecules bind to the composite surface through a combination of electrostatic interactions with protonated amine groups and hydrophobic association of the perfluoroalkyl chains with the polymer and lignocellulosic domains.

**Figure 4:**
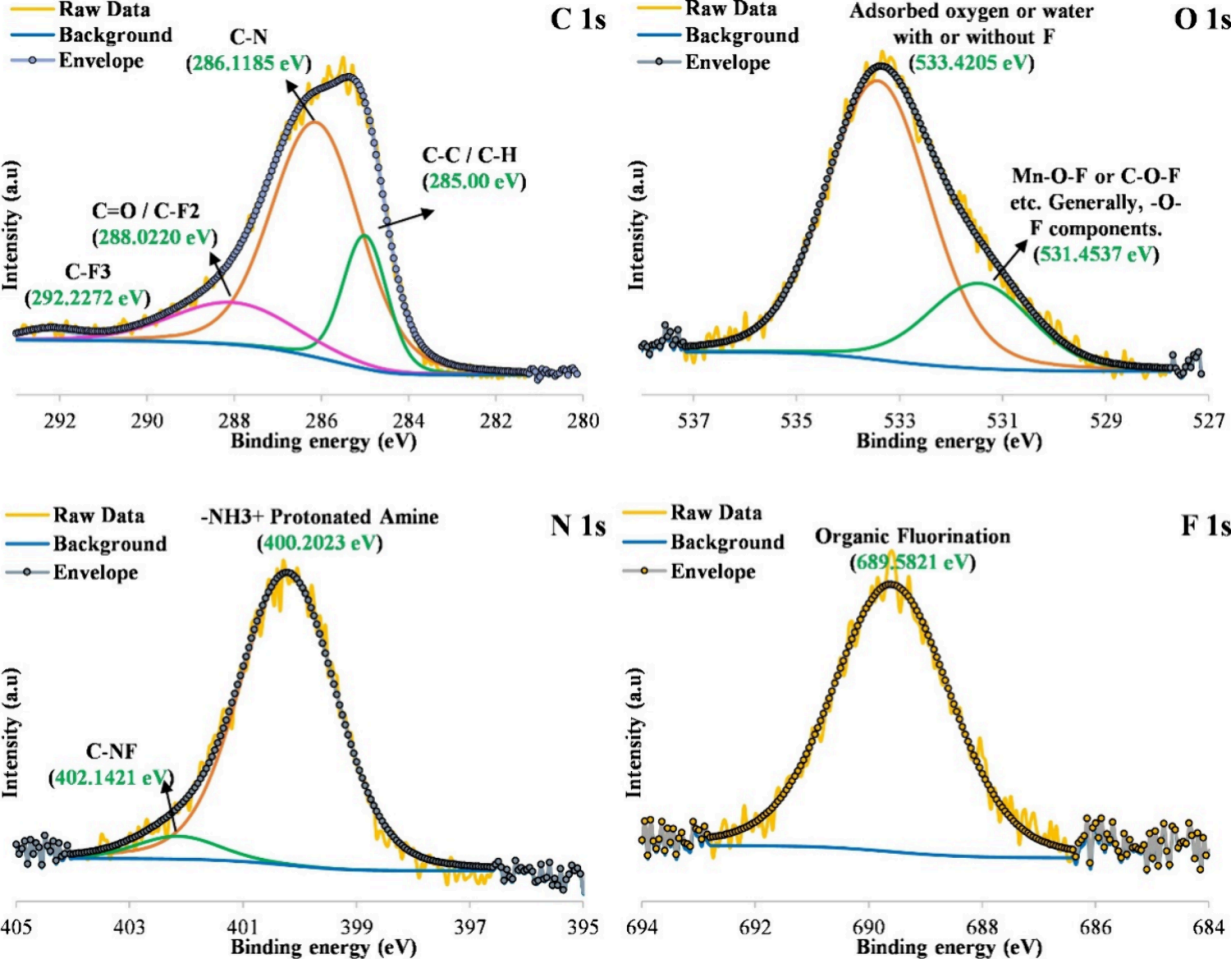
XPS survey of sawdust/MnO_2_/PmPD after PFAS adsorption.

Figure 5 exhibits the surface morphology of the adsorbent before and after PFAS adsorption. As can be seen, the structure of the adsorbent resembled the agglomeration of numerous spherical sites. After adsorption, this structure became more homogeneous and less granular, suggesting the interactions with PFAS molecules in the aqueous solution and surface coverage by adsorbed species.

**Figure 5:**
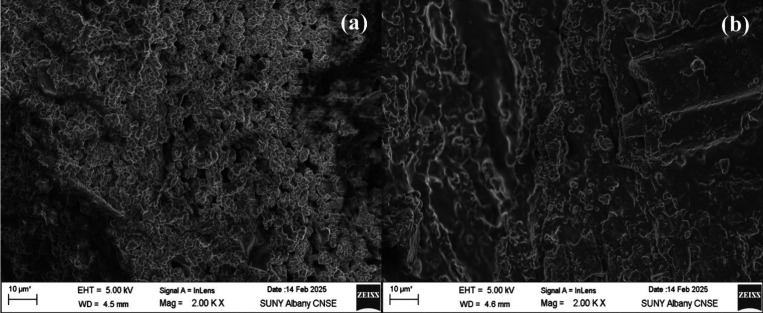
SEM results of sawdust/MnO_2_/PmPD (a) before adsorption and (b) after adsorption.

Moreover, EDX analysis was performed to gather information about the elemental composition of the prepared sorbent. As shown in Figure S7, C (58.99%), O (32.09%), and N (6.83%), were the main elements of the synthesized adsorbent. After adsorption, EDX scans showed the appearance of F^–^ (0.92%), which demonstrated PFAS adsorption to the surface of the sawdust/MnO_2_/PmPD. Because EDS has a relatively low sensitivity to light elements and is not suitable for quantitative fluorine determination, these results are interpreted only as qualitative evidence of F incorporation. All details can be found in Table S8.

### 3.5 Effect of pH

The pH of a solution can affect the electrostatic interactions between an adsorbent and adsorbates.^39^ It is known that when the solution pH is below the value of an adsorbent’s ZPC, the surface charge of the adsorbent is positive. To investigate the pH effect, different PFAS solutions were prepared with pH values of 4.0, 5.7, 7.0, 8.0, and 11. Other factors, such as contact time, adsorbent dose, and PFAS initial concentrations, were kept unchanged. As shown in Figure 6a, the highest removal efficiency was observed with pH 5.7, and there was a slight decrease when the pH was 7.0 and 8.0 (except for GenX). Increasing the pH to 11, however, led to a significant decrease in the removal of PFBS, PFHxA, PFHpA, and GenX. For long-chain PFAS, the drop in removal efficiency was not as significant as that for the short chains. These results are consistent with the ZPC analysis, as the highest zeta potential was at pH 6 and the lowest was 12 (Figure 6b).

**Figure 6:**
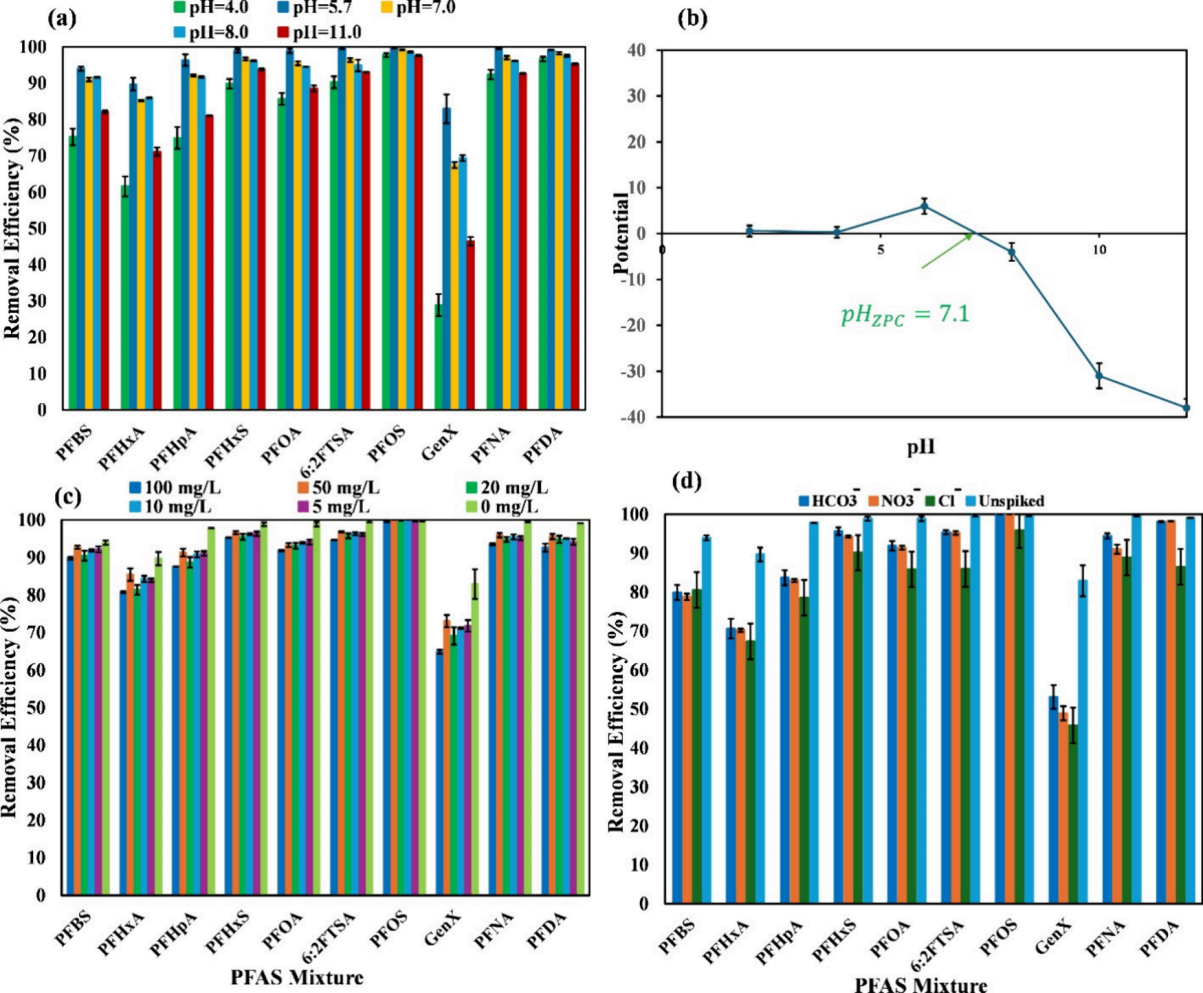
(a) Effect of pH on PFAS removal, (b) ZPC analysis of sawdust/MnO_2_/PmPD, and (c, d) effect of natural organic matter and coexisting anions on PFAS removal, respectively.

### 3.6 Effect of Natural Organic Matter

It is well-known that water in real environments contains natural organic matter (NOM).^40^ This substance comprises aliphatic and aromatic regions and an abundance of carboxyl groups in its structure, affecting the performance of an adsorbent when they are copresent with other pollutants such as PFAS. To determine the possible effect of NOM on PFAS removal, humic acid (HA) was selected as a representative for NOM in the concentration range of 0, 5, 10, 50, and 100 mg L^–1^. This range is representative of NOM in natural surface water.^41^ As indicated by Figure 6c, compared to HA at 0 mg/L, HA at 5 mg/L led to decreased PFAS removal of all but PFOS. In the range of HA from 5 to 50 mg/L, the decrease in removal was not significant. At the highest HA concentration of 100 mg/L, the drop in the level of sorption was significant for most PFAS. Again, PFOS was an exception. These observations were consistent with the assumption that HA with a negative charge competed with PFAS for the positively charged active sites on sawdust/MnO_2_/PmPD. Following the adsorption of NOM on the adsorbent, due to the accumulation of negative charges, the potential surface charge of the adsorbent will drop and, under extreme conditions, exert repulsion to negatively charged PFAS compounds, leading to a decrease in the removal efficiency.^42^ As can be seen in the plot, the drop in the removal efficiency was less and more pronounced for long and short chains, respectively. This observation suggests HA had a minor effect on the hydrophobic sites of the adsorbent and could not disrupt the long-chain PFAS adsorptions.^43^ On the other hand, since short-chain PFAS typically relies on electrostatic interactions with adsorbent rather than hydrophobic interactions, following the reduction in surface charge, their adsorption became more sensitive to the presence of HA and eventually dropped. Overall, sawdust/MnO_2_/PmPD still maintained its strong sorption of most PFAS when the concentration of HA was less than 50 mg/L.

### 3.7 Effect of Coexisting Anions

It is recognized that ions in a solution can shield the positive charges on an adsorbent, weakening the potential electrostatic interactions between the adsorbent and adsorbates. Additionally, ions can compress the electrical double-layer,^44^ which likely leads to a decrease in the removal of PFAS, since most of which are negatively charged in a solution with a neutral pH. As shown in Figure 6d, all spiked ionic species, bicarbonate, nitrate, and chloride, each at 1 mM, had a similar but significantly negative effect on the removal of all target PFAS. The only exception was PFOS and PFNA, the most hydrophobic ones among the target ten PFAS, for which the negative effect from bicarbonate and nitrate was not too significant.

The negative effect on the removal of less hydrophobic PFAS could be due to the spiked high concentration of anions. For example, 1 mM nitrate is 62 mg NO_3_/L and has 14 mg N/L. As shown in Table S4, the total N concentration in the Hudson River sample was 1.53 mg/L, and the nitrate concentration was less than the detection limit. Considering that the maximum contaminant level (MCL) for nitrate in drinking water set by the EPA is 10 mg/L (mg/L), if the sawdust/MnO_2_/PmPD is used to remove PFAS in drinking water, then the presence of anions, including nitrate, may not lead to decreased performance of the sorbent. This will be explored in our future studies.

The anion’s negative impact on PFAS removal did point out the existence of electrostatic interactions between PFAS and sawdust/MnO_2_/PmPD, and the competition between the anions and negatively charged PFAS in occupying the positively charged sites. Thus, if sawdust/MnO_2_/PmPD is used to remove PFAS in water with a high ionic strength, the sites with positive charges must be increased. This calls for modification of or re-engineering of the material to attain different properties for different applications.

### 3.8 Regeneration and Reuse of the Adsorbent

With a hydrophobic tail and hydrophilic head functional group, PFAS are amphiphilic and bind to sawdust@MnO_2_@PmPD through both hydrophobic and electrostatic interactions. To regenerate the PFAS-laden sawdust@MnO_2_@PmPD, a regenerant must be able to disrupt both interactions. 1% methanolic ammonium hydroxide was tested to be a suitable regenerant that led to almost 100% removal of PFAS from the spent adsorbent. This basic methanol is a required reagent for extracting PFAS from different sample matrices, such as soil, biosolids, and tissue samples, as shown in EPA Method 1633. Theoretically speaking, methanol has the potential to weaken the hydrophobic interactions between the backbone of the adsorbed PFAS and the adsorbent, and salts such as ammonium hydroxide can disrupt the electrostatic interactions and replace PFAS molecules with anions of hydroxide.^45^

As shown in Figure 7a, almost all PFAS were desorbed. This methanol rinse, however, dissolved part of PmPD from the spent sorbent. This was reflected in the decreased capture of PFAS when the rinsed sorbent was reused in cycle #2 (Figure 7b). Upon soaking the rinsed sorbent back into the spent mPD solution, which was used initially to synthesize the sawdust/MnO_2_/PmPD, the sorbent’s ability to remove PFAS was recovered. This was demonstrated by a similar PFAS removal efficiency for the sorbent used in the first, third, fourth, and fifth cycles.

**Figure 7:**
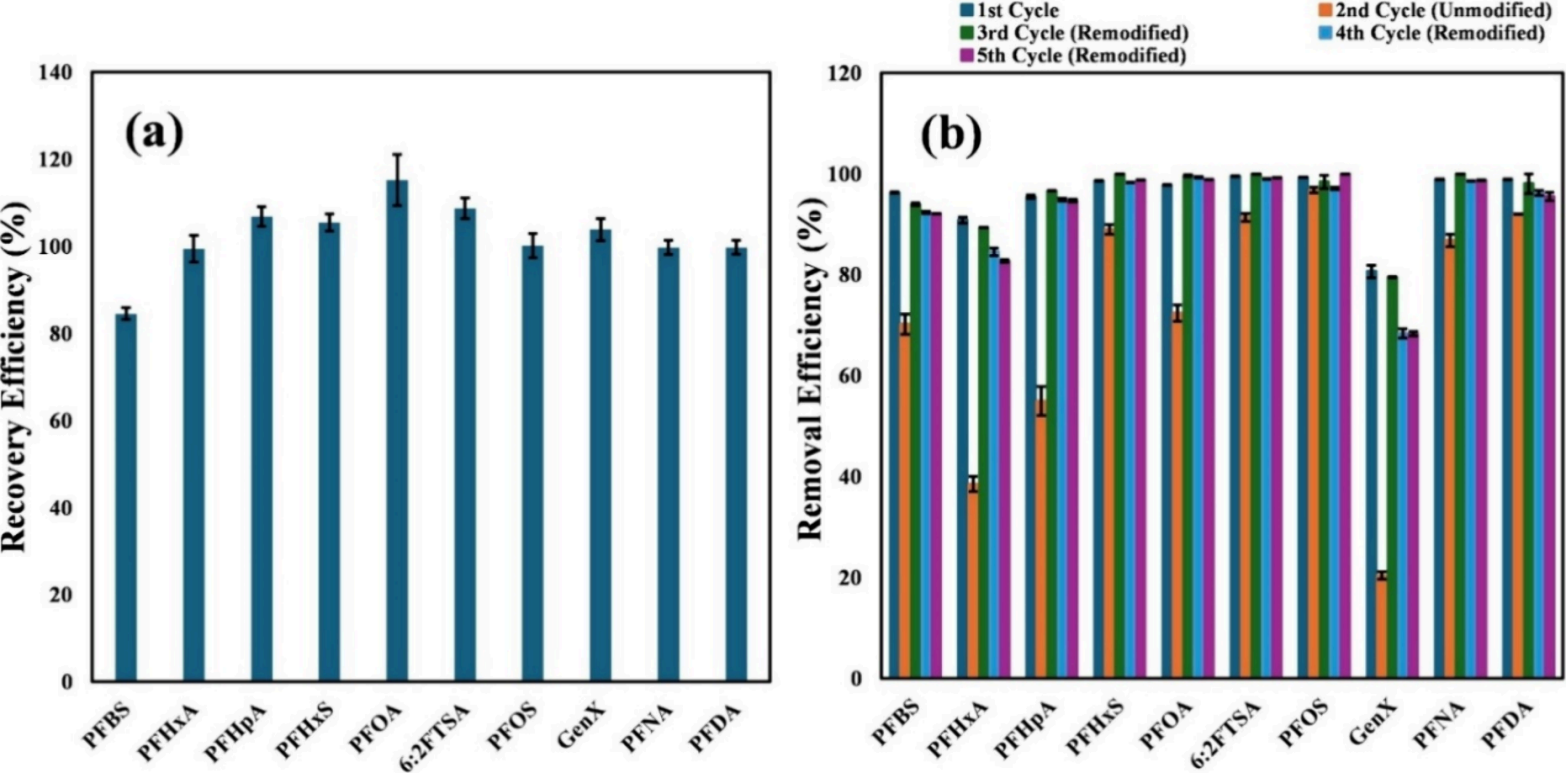
(a) Recovery of PFAS from the spent sorbent by basic methanol rinse. (b) Reusability with or without remodification of the adsorbent.

To understand the regeneration process better, the remodified sawdust/MnO_2_/PmPD was subjected to analysis by SEM/EDX, and FT-IR. As shown in Table S8 and Figure S8, the remodified adsorbent contained 6.6% of N, which was similar to that in the preadsorption material.

FT-IR (Figure S9) revealed the typical bands of MnO_2_ (600 cm^–1^) and NH_2_ functional groups (3306 cm^–1^). Thus, these analyses confirmed that MnO_2_ particles and PmPD remained attached to the adsorbent’s surface through the regeneration process, and the desorbed sorbent could be regenerated and reused for at least five cycles.

Solvent-based regeneration approaches are often considered environmentally concerning due to the generation of secondary liquid waste containing desorbed contaminants. Several approaches can be adopted to mitigate this issue and minimize possible environmental impacts. These approaches include, but are not limited to, (1) destruction of PFAS in the concentrated waste through UV/sulfite photoreduction, heat-activated persulfate oxidation, and ozonation;^46,47^ (2) injection to underground wells as described in the published interim guidelines for the destruction and disposal of PFAS compounds by the US EPA, Section 3.c “Underground Injection”.^45^ This method, however, is under development and requires further research and analysis to determine the fate and transport of PFAS in the subsurface as it depends on the physicochemical properties of the discharged PFAS, and the geochemical properties of injection zones; and (3) disposal at hazardous landfills. This is the least ideal option, but commonly practiced for PFAS-containing materials. For the concentrated waste generated from the regeneration of spent sawdust@MnO_2_@PmPD, at least these three options will be explored later to arrive at the best approach for waste disposal.

### 3.9 River Water Studies

To investigate whether the sawdust/MnO_2_/PmPD was able to remove PFAS from real environmental water, samples from the Hudson River in Albany, New York, were collected. As seen in Figure S10, there was a slight decrease in the removal of long-chain PFAAs and 6:2 FTSA. The capture of short-chain PFAAs (i.e., PFHxA, PFHpA, and PFBS) and GenX, however, decreased considerably. This drop could be due to (1) the presence of organic compounds and F^–^ anions detected in the water samples. The measured TOC and F^–^ were around 3.53 and 0.43 mg/L, respectively (Table S4). These species are known as competitors of PFAS for occupying the active sites on the surface of the adsorbent,^48^ (2) pH—the river water had a pH of 8.37, which was higher than the optimal pH of 5–6 for PFAS capture, and (3) the possible existence of other ions and compounds that we did not measure. Overall, although less than 100% of the spiked PFAS were removed by sawdust/MnO_2_/PmPD, this material presented itself as a low-cost candidate for capturing >90% of long-chain PFAS in the collected river water.

Rivers and streams’ water quality varies across the US in terms of location and seasonal change. Typically, surface water contains a wide range of cations, anions, and natural organic matter. It is not possible to study natural water from different water bodies in this study. The use of the Hudson River samples was to prove the concept that the sawdust@MnO_2_@PmPD material can be adopted for removing PFAS in such water. If water in other places is more turbid or has higher concentrations of ions and other pollutants, then pretreatment may be needed to facilitate effective PFAS capture.

## 4 Proposed Mechanisms

As discussed above, the raw sawdust had poor sorption, and sawdust/MnO_2_ had limited sorption of PFAS in water. The impressive PFAS removal by sawdust/MnO_2_/PmPD indicated the indispensable role of PmPD in the sorption process. As revealed by sorbent characterization above, the presence of PmPD on sawdust/MnO_2_’s surface provided positive charges and hydrophobicity to allow electrostatic and hydrophobic interactions between PFAS and the sorbent (Figure 8). Based on the XPS analysis, the shifts in the N element reveal the major role of amine groups of PmPD in PFAS adsorption via the negative polar head of PFAS and protonated NH_3_^+^. Considering the conjugated benzene rings of PmPD, this structure can provide hydrophobic sites to interact with the nonpolar regions of PFAS (C–F chains) and facilitate the removal process. Moreover, Mn–O functional groups of the adsorbent also contributed to the PFAS adsorption, most likely due to van der Waals interactions between the positive pole of Mn in the Mn–O structure. However, this contribution was less pronounced since the binding energy of the Mn–O functional groups showed a minimal change in the XPS spectrum. These interactions were strong enough to tolerate the change of pH, the presence of HA, and coexisting anions. Aside from these two types of interactions, hydrogen bonding cannot be excluded as the carboxyl and sulfonyl head of PFAS can form hydrogen bonds with NH_2_ functional groups. This was proven by the XPS and FT-IR analyses.^49^

**Figure 8:**
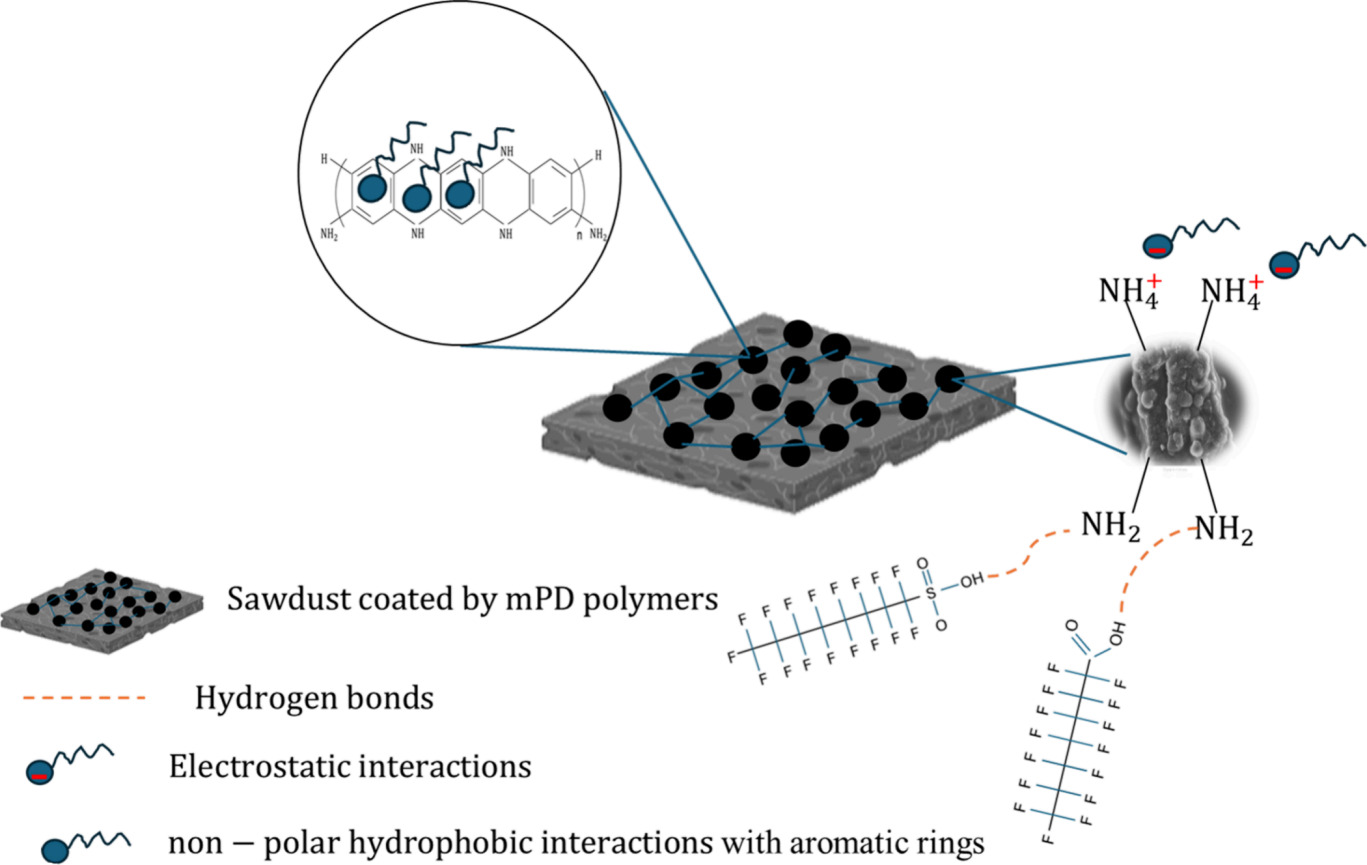
Proposed adsorption mechanisms involved in capturing PFAS by the sawdust/MnO_2_/PmPD.

## 5 Conclusions

Here, we report an extremely simple synthesis process for fabricating sawdust/MnO_2_/PmPD. It is worth noting that one of the central goals of this study was to avoid carbonization and pyrolysis of feedstocks (such as straw and wood chips) to acquire chars or activated carbons (ACs). As literature suggests, the preparation of AC demands physical and chemical activation and subjecting the activated feedstock to elevated temperatures.^50^ These steps consume a considerable amount of chemical substances and energy, which leads to the complexity of the synthesis process. In this study, as detailed above, the sawdust did not undergo the aforementioned steps and was used directly in the polymerization of mPD. Besides the sorbent’s over 90% of removal of the 10 target PFAS, this material can be regenerated easily and reused for at least five cycles. The low cost of sawdust/MnO_2_/PmPD is justified by the inexpensiveness of sawdust itself, the low-temperature synthesis procedure, and the repeated use of the original mPD solution for sorbent regeneration. Although the removal of short-chain PFAS was affected by environmental factors, the capture of long-chain PFAS remained steady and strong regardless of change of pH, increasing concentration of HA, and the coexistence of high concentrations of cations and anions. Given the fast kinetics and the ability of the material to capture a mixture of PFAS at the low end of ppb levels, the sawdust/MnO_2_/PmPD holds promise to be used in large scale for removing PFAS in real surface water at least. The availability of this inexpensive material could also open doors for in-depth environmental science and management studies and the use of sawdust-based sorbents for capturing PFAS in the water environment and beyond. It needs to be noted that detailed technoeconomic analysis and life cycle analysis will need to be performed for the sawdust/MnO_2_/PmPD in comparison with existing PFAS removing materials before the reported sorbent can be adopted on an industrial scale. Additionally, given the variability of sawdust generated from different sources and process methods, whether the same synthesis procedure reported here can be applicable to other types of sawdust remains to be elucidated. This further elucidation will allow an in-depth analysis regarding the reproducibility and scalability of the whole process.

## Supplementary Material



## Notes

Additional experimental details, materials, methods, and data analysis presented in the form of text, table, and figure (PDF)
