# Supplementary material for: Fast Adsorption of Short and Long-Chain Per- and Polyfluoroalkyl Substances from Water by Chemically Modified Sawdust
Source: ACS ES T Water. 2026 Jan 28;6(2):873–83. doi: 10.1021/acsestwater.5c00960 (PMC12910595; doi:10.1021/acsestwater.5c00960)
Supplement: Supplementary file 1 [file ew5c00960_si_001.pdf]

Supporting Information

Fast adsorption of short and long-chain PFAS from water by chemically modified sawdust

Behnia Bitaraf[a], Md. Nahid Pervez[a*], Tao Jiang[a], Mariana Maria Ioanniti[b], Haralabos Efstathiadis[b], Mehmet V. Yigit[c], Yanna Liang[a*]

[a]*Department of Environmental and Sustainable Engineering, University at Albany, State University of New York, Albany, NY 12222, USA*
[b]*Department of Nanoscale Science and Engineering, University at Albany, State University of New York, Albany, NY 12222, USA*
[c]*Department of Chemistry, University at Albany, State University of New York, Albany, NY 12222, USA*

* Corresponding authors:

mpervez@albany.edu (M.N. Pervez)
yliang3@albany.edu (Y. Liang)

Text S1. PFAS Analysis

In order to analyze and quantify the studied PFAS, at different time points during adsorption, 1 mL of sample was withdrawn, centrifuged at 12,000 rpm for 15 min, and 0.4 mL of the supernatant were transferred into LC/MS vials. A 1290 Infinity II LC system and a 6470 Triple Quad Mass Spectrometer (LC-MS/MS, Agilent Technologies, Santa Clara, CA, USA) were used to quantify the target PFAS in the samples. Two Agilent Eclipse Plus C18 columns were applied in this study: 1) a delay column that measured 4.6 × 50 mm and had a particle size of 3.5 µm, and a ZORBAX analytical column that was 3 × 50 mm and had a particle size of 1.8 µm. The temperature of the columns was set at 50°C. Ammonium acetate with a concentration of 5 mM, and 95% methanol were used as the binary mobile phase solvents A and B, respectively. The flow rates of the abovementioned solvents were adjusted at 0.5 milliliters per min. The initial composition of the solvents, 70% A and 30% B, turned to 0% A and 100% B after 8 minutes, and it was maintained at this combination for 4 minutes before going back to the initial settings. The entire process took 15 min. More details are provided in our previous publications [1]. The dynamic multiple reaction monitoring transitions and limit of detection (LOD) of each PFAS were given in Table S3.

Text S2. Chemical analysis

To measure the concentration of PFAS in the Hudson River, the EPA method 537.1 was followed. In this manner, a surrogate of 10 μL PFHxS (10 ng, 1 mg L^{-1}) was introduced to each 400 mL sample. Thereafter, the spiked sample was passed through a Hypersep C18 cartridge conditioned with deionized water and methanol. Once all 400-mL sample passed through the cartridge, PFAS retained on the C18 cartridge were eluted and then spiked with ^{13}C-PFOS and ^{13}C-PFOA as internal standards. Following the use of the Agilent LC-MS/MS, the targeted PFAS were quantified. Further information about the application of LC/MS method can be found in the previous **Text S1**. For analyzing and detecting anions in the Hudson River water, a 930 Compact IC Flex instrument was used. The anions' separation was accomplished through a Metrosep SUPP 5 column. The flow rate was 0.7 mL/min. The mobile phase was a 1:1 mixture of Na_2CO_3 at 1 mM and 1.7 mM $NaHCO_3$. After that, 0.05 M sulfuric acid was used as a regeneration agent in order to decrease the conductivity. Calibration curves were obtained in the range of 1 to 500 μg L^{-1} using the standard mixture (Thermo Fisher Scientific Inc., USA), which contains Cl^-, F^-, Br^-, SO_4^{2-}, NO_3^- , PO_4^{3-}. Finally, to investigate the concentration of TOC and TN in the river water, the Shimadzu TOC-L analyzer (Columbia, MD), and TNT 828 kit were utilized, respectively.

Table S1. List of materials used in this study

Materials	Grade/Purity	Supplier
Sawdust	-	Shannon's sawmill
$KMnO_4$		Cole-Parmer
mPD	99%	Sigma Aldrich
Water	LC/MS Grade	Fisher Scientific
Methanol	LC/MS Grade	Fisher Scientific
Ammonium hydroxide	28-30%	Fisher Scientific
Perfluorohexanoic acid	≥ 98%	Frontier Scientific
Perfluoroheptanoic acid	≥ 98%	Matrix Scientific
Perfluorooctanoic acid	≥ 96%	Sigma-Aldrich
Perfluorononanoic acid	≥ 98%	Oakwood Chemicals
Perfluorodecanoic acid	≥ 98%	Matrix Scientific
Potassium perfluorobutanesulfonate	≥ 98%	Frontier Scientific
perfluorohexanesulfonic acid	≥ 98%	Fisher scientific
Heptadecafluorooctanesulfonic acid	≥ 98%	Sigma-Aldrich
Undecafluoro-2-methyl-3-oxahexanoic acid	≥ 97%	SynQuest Laboratories
6:2 Fluorotelomer sulfonic acid	≥ 97%	SynQuest Laboratories

Table S2. Physicochemical properties of PFAS used in this research.

Category	Compound	Abbreviation	Chemical formula	Molecular weight (g/mol)	S_w (25 °C)	pK_a (25 °C)
PFOS alternative	6:2 Fluorotelomer sulfonic acid	6:2 FTSA	$C_8H_5F_{13}O_3S$	428	1.3 [1]	1.31 [2]
Short-chain PFSA	Potassium perfluorobutanesulfonate	PFBS	$C_4F_9O_3SK$	338	46.2 [2]	0.14 [3]
Long-chain PFSA	Perfluorohexanesulfonic acid potassium salt	PFHxS	$C_6F_{13}KO_3SK$	438	2.3 [1]	0.14 [3]
Long-chain PFSA	Heptadecafluorooctanesulfonic acid potassium salt	PFOS	$C_8HF_{17}KO_3SK$	538	0.57 [4]	-3.27 [4]
Short-chain PFCA	Perfluorohexanoic acid	PFHxA	$C_6HF_{11}O_2$	314	15.7 [4]	-0.16 [3]
Short-chain PFCA	Perfluoroheptanoic acid	PFHpA	$C_7HF_{13}O_2$	364	3.65 × 10^{-3} [6]	-2.29 [5]
Long-chain PFCA	Perfluorooctanoic acid	PFOA	$C_8HF_{15}O_2$	414	3.4 [4]	-0.2 [3]
Long-chain PFCA	Perfluorononanoic acid	PFNA	$C_9HF_{17}O_2$	464	6.25 × 10^{-2} [6]	-0.21 [5]
Long-chain PFCA	Perfluorodecanoic acid	PFDA	$C_{10}HF_{19}O_2$	514	9.5 [1]	-5.2 [6]
PFOA alternative	Undecafluoro-2-methyl-3-oxahexanoic acid	GenX	$C_6HF_{11}O_3$	330	N/A	2.84 [6]

Note: Sw: solubility in water, g/L; pKa: dissociation constant; N/A: data not available.

Table S3. Dynamic multiple reaction monitoring transitions and limit of detection (LOD) of the PFAS tested in this study.

**Compound name**	**Retention time (min)**	**Precursor ion**	**Quantification ion mass**	**Confirmation ion mass**	**Limit of detection (ng/L)**
**PFBS**	3.6	299	99	80	2.4
**PFHxS**	5.7	399	99	80	1.2
**PFHxA**	4.67	313	269	119	2.0
**PFHpA**	5.63	363	319	169	4.1
**GenX**	4.9	285	185	169	4.1
**PFOA**	6.3	413	369	169	1.0
**PFOS**	6.84	499	99	80	1.7
**PFNA**	6.8	463	419	169	5.2
**PFDA**	7.24	513	469	218.7	2.1
**6:2 FTSA**	6.56	427	406.8	79.9	10.6

Note: N/A: data not available

Table S4. Characteristics of the Hudson River water used in this study.

Parameter	Value
pH	8.27 ± 0.12
TN (mg/L)	1.53 ± 0.21
TOC (mg/L)	3.53 ± 0.09
F^- (mg/L)	0.41± 0.01
Cl^- (mg/L)	ND
SO_4^{2-} (mg/L)	ND
NO_3^- (mg/L)	ND
PFNA (ng/L)	3.75 ± 1.23
PFDA (ng/L)	7.93 ± 2.90

Note: TN: total nitrogen; TOC: total organic carbon; ND: not detected.

Table S5. Parameters and values derived from adsorption isotherm models of Langmuir, Freundlich, Sips, and Toth for total PFAS.

Model	Parameter	Value
Langmuir	R^2	0.9755
	Adjusted R^2	0.9673
	K_L (L/mg)	7.44×10^{-5}
	q_m (mg/g)	97.14
Freundlich	R^2	0.9787
	Adjusted R^2	0.9716
	K_F (mg×L$^{1/m}$/(g×mg$^{1/m}$))	4.73×10^{-3}
	m	0.93
Sips	R^2	0.9849
	Adjusted R^2	0.9798
	K_S (L/mg)	5.02×10^{-3}
	q_m (mg/g)	2.93
	n	0.62
Toth	R^2	0.9617
	Adjusted R^2	0.9490
	K_T (L/mg)	4.23×10^{-3}
	q_m (mg/g)	1.89
	t	2.90

Table S6. Experimental and isotherm modeled values of total adsorbed PFAS at equilibrium (q_e) and concentrations in aqueous phase at equilibrium (C_e) in the adsorption process by sawdust@MnO_2@PmPD

∑PFAS C_e (μg/L)	∑PFAS q_e experimental (mg/g)	∑PFAS q_e modeled (mg/g)			
		Langmuir	Freundlich	Sips	Toth
6.09	0.13	0.04	0.03	0.01	0.05
45.83	0.16	0.33	0.29	0.25	0.37
55.73	0.40	0.40	0.36	0.33	0.44
123.06	0.92	0.88	0.85	0.92	0.94
219.20	1.57	1.56	1.59	1.57	1.43

Table S7. Summary of binding energy and atomic concentration of sawdust@MnO_2@PmPD

Samples	Atomic Concentration					
	C 1s	F 1s	N 1s	O 1s	Cl 2p	Mn
Before Adsorption	77.48%	-	13.05%	8.24%	1.22%	Trace
After Adsorption	71.06%	4.31%	9.79%	14.84%	-	Trace
Remodified	79.89	-	12.33%	5.56	2.21%	-
Samples	Binding Energy (eV)					
	C 1s	F 1s	N 1s	O 1s	Cl 2p	
Before Adsorption	283.00	-	397.50	530.50	196.00	
After Adsorption	283.50	688.00	398.00	531.50	-	
Remodified	283.50	-	12.33%	531.00	195.00	

Table S8. Summary of the elemental composition of sawdust@MnO_2@PmPD EDX analysis.

Before Adsorption

Element	series	Unn .C (wt%)	Norm. C (wt%)	Atom. C (at%)	3 Sigma (wt%)
Carbon	K- series	58.94	58.94	66.20	18.41
Nitrogen	K- series	7.30	7.30	7.12	2.74
Oxygen	K- series	29.95	29.95	25.25	9.65
Magnesium	K- series	0.01	0.01	0.01	0.08
Sodium	K- series	0.07	0.07	0.04	0.09
Chlorine	K- series	3.62	3.62	1.38	0.45

After Adsorption

Element	series	Unn .C (wt%)	Norm. C (wt%)	Atom. C (at%)	3 Sigma (wt%)
Carbon	K- series	58.99	58.99	65.6	18.42
Nitrogen	K- series	6.83	6.83	6.52	2.58
Oxygen	K- series	32.09	32.09	26.8	10.33
Magnesium	K- series	0.00	0.00	0.00	0.00
Fluorine	K- series	0.92	0.92	0.65	0.45
Sodium	K- series	0.01	0.01	0.01	0.08
Chlorine	K- series	1.15	1.15	0.43	0.20

Remodified Adsorbent

Element	series	Unn .C (wt%)	Norm. C (wt%)	Atom. C (at%)	3 Sigma (wt%)
Carbon	K- series	61.95	61.95	69.75	19.33
Nitrogen	K- series	6.60	6.60	6.37	2.47
Oxygen	K- series	25.58	25.58	21.62	8.27
Magnesium	K- series	0.00	0.00	0.00	0.00
Sodium	K- series	0.09	0.09	0.05	0.09
Chlorine	K- series	5.76	5.76	2.20	0.67

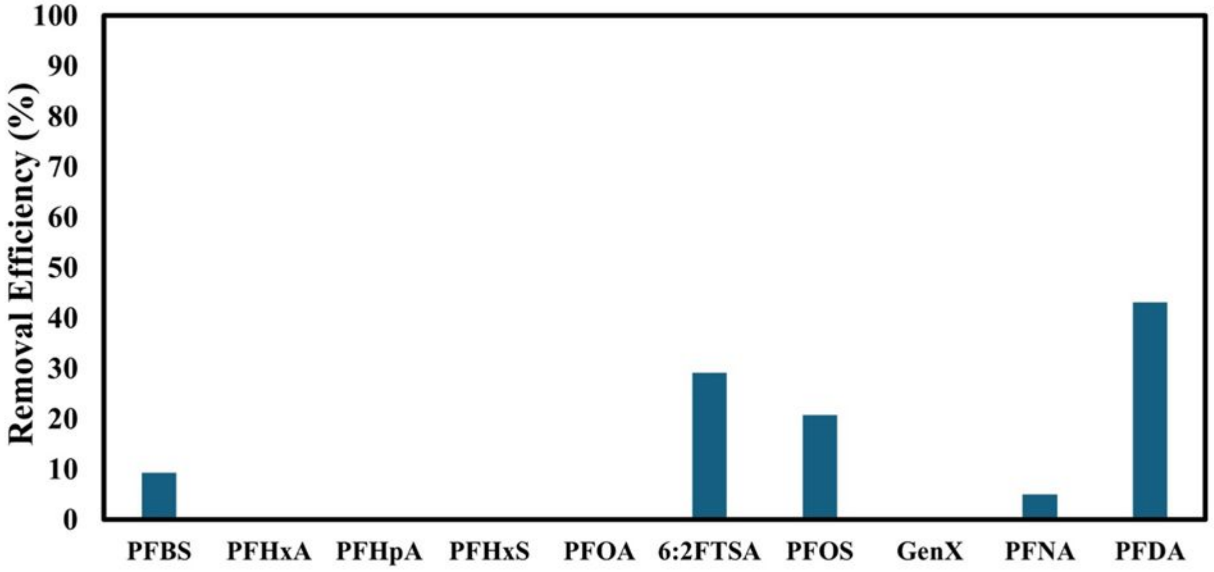

Figure S1. The removal efficiency of sawdust/MnO_2.

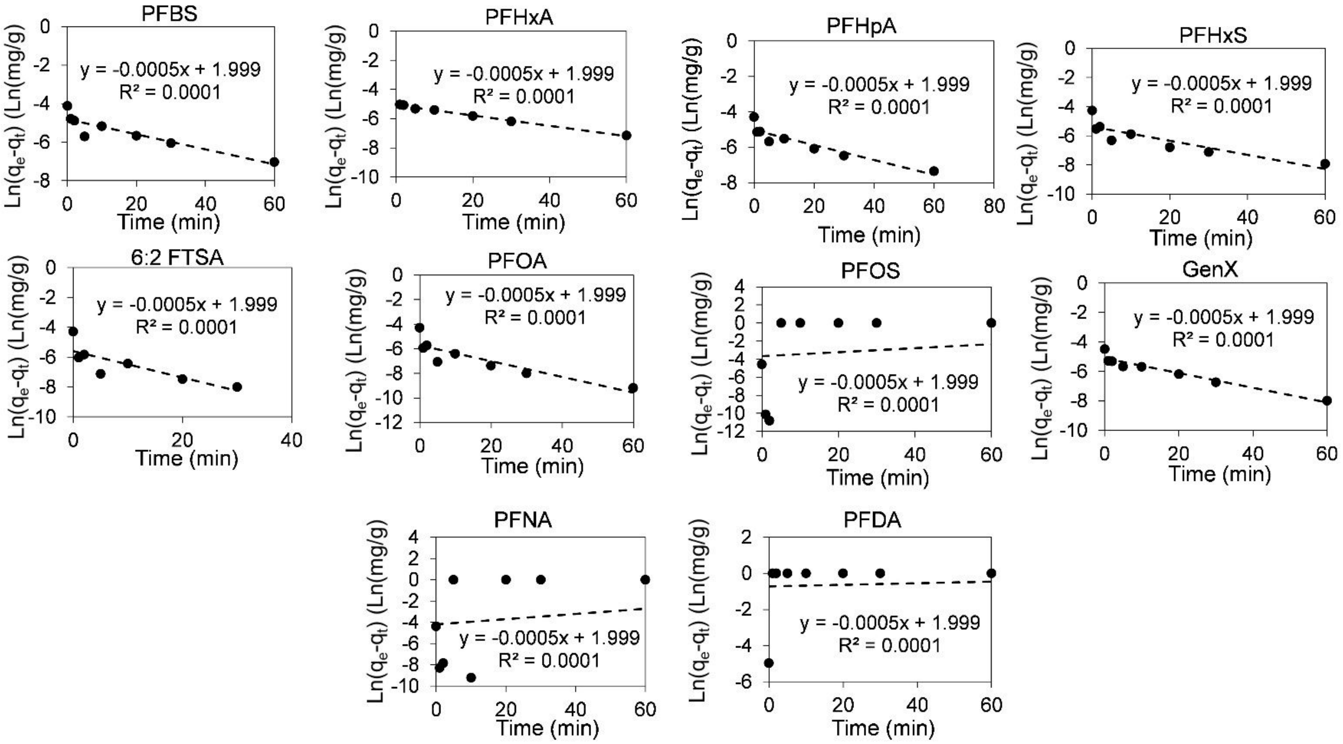

Figure S2. Linear form fitting of the pseudo-first-order model of the PFAS adsorption process with the initial concentration of 10 μg L^{-1}.

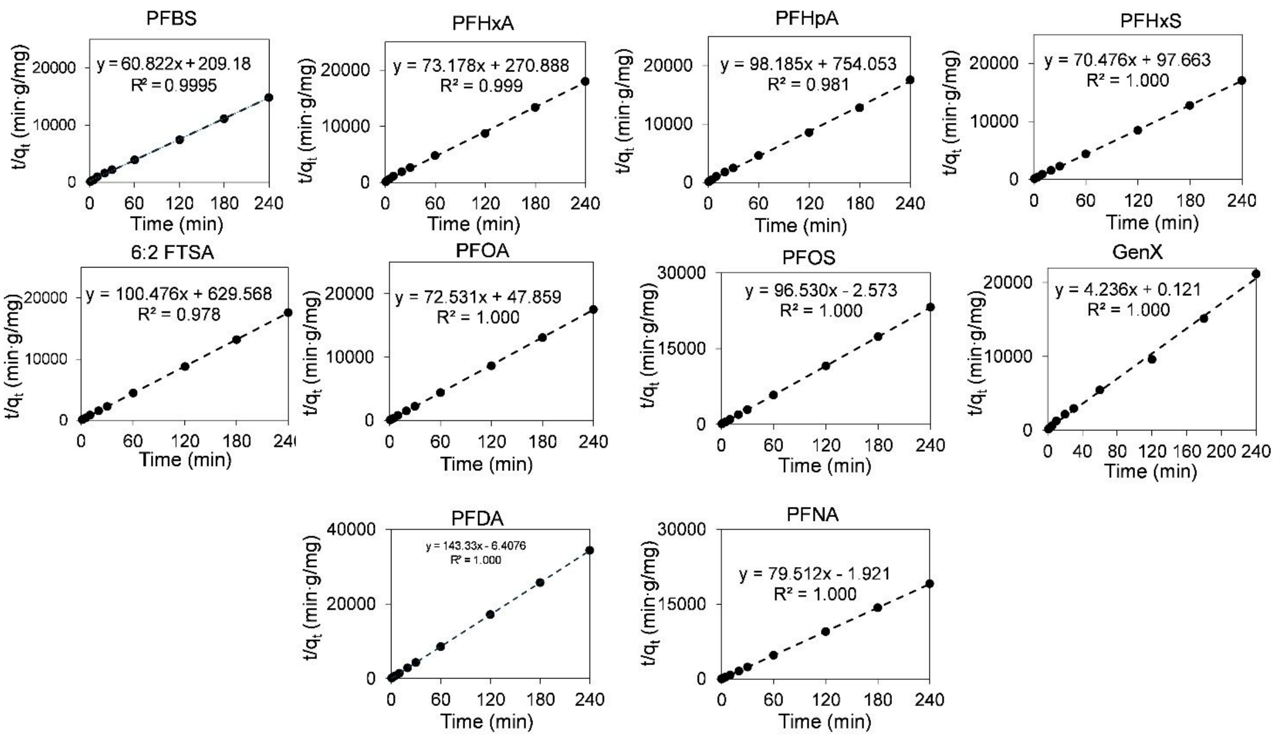

Figure S3. Linear form fitting of the pseudo-second-order model of the PFAS adsorption process with the initial concentration of 10 μg L^{-1}.

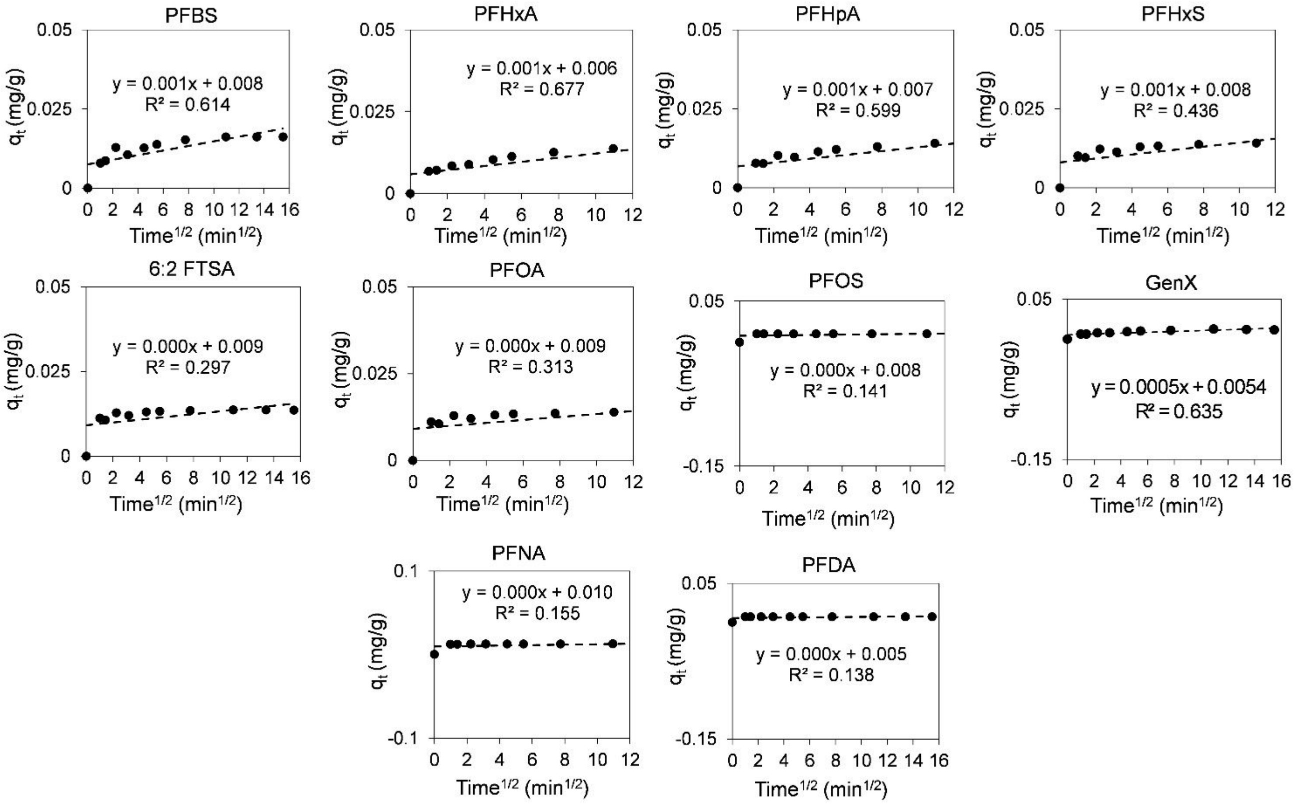

Figure S4. Interparticle diffusion model of the PFAS adsorption process with the initial concentration of 10 µg L^{-1}.

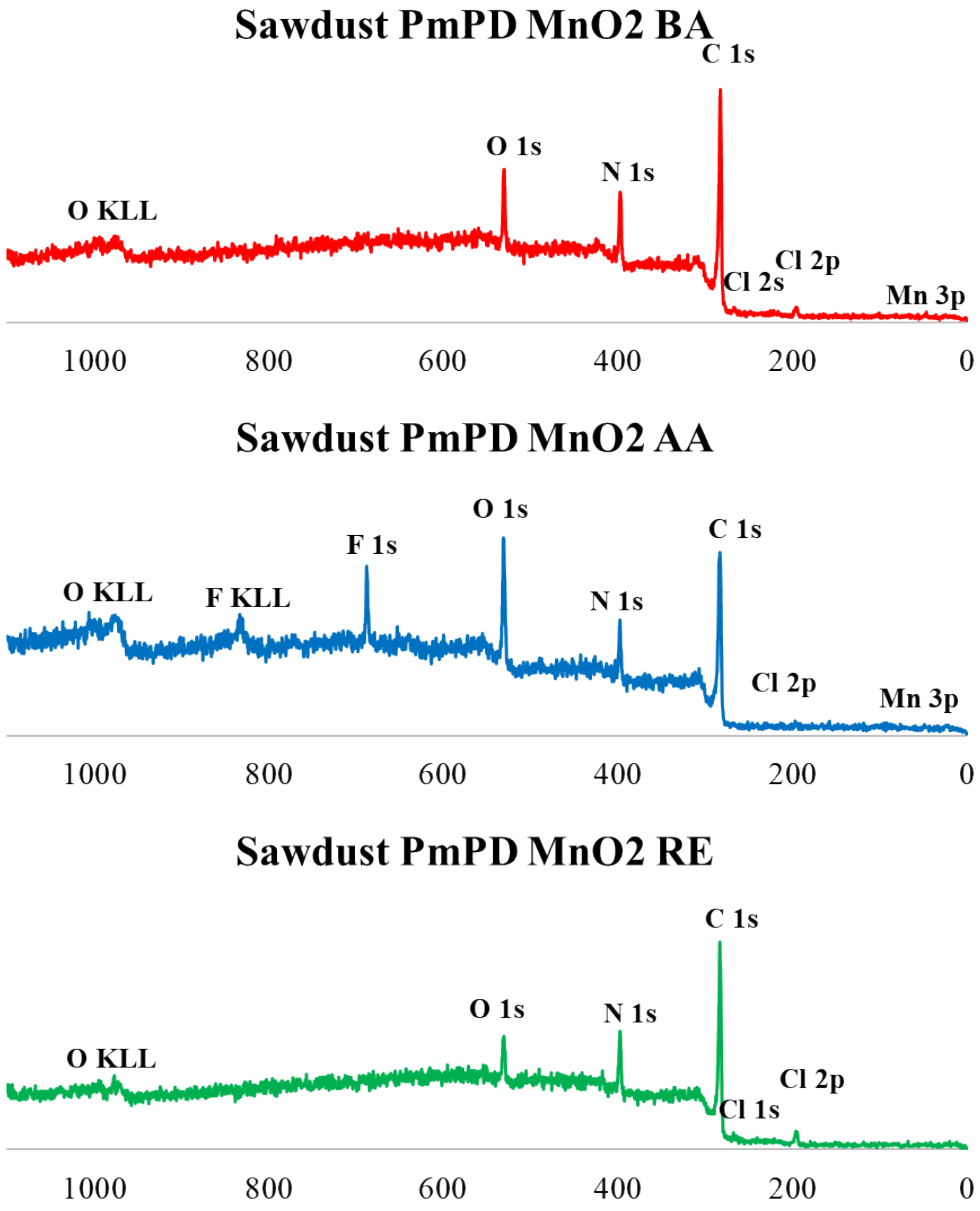

Figure S5. XPS survey of sawdust@MnO_2@PmPD. BA: before adsorption, AA: after adsorption, RE: after regeneration.

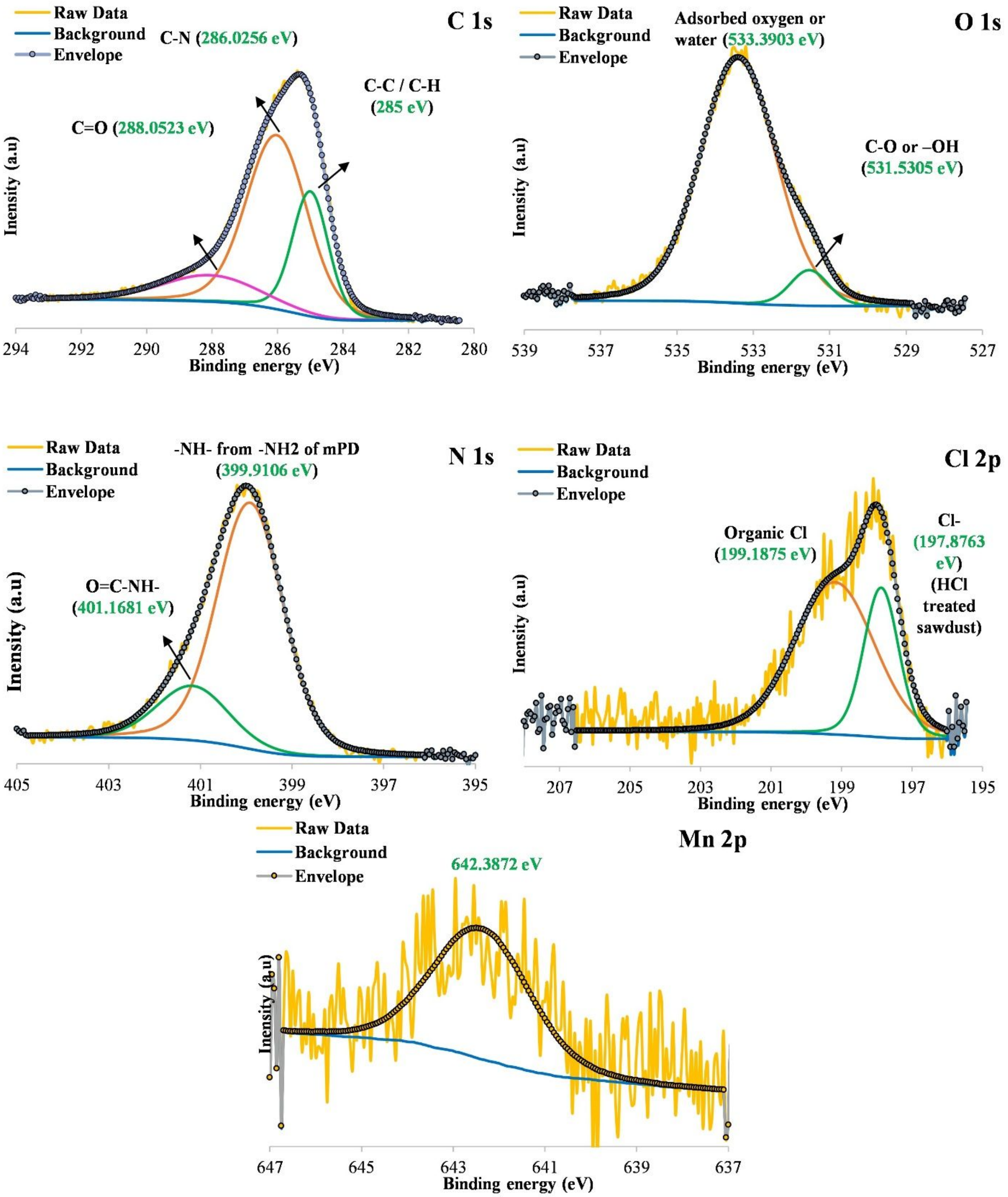

Figure S6. XPS survey of sawdust@MnO_2@PmPD before adsorption (BA).

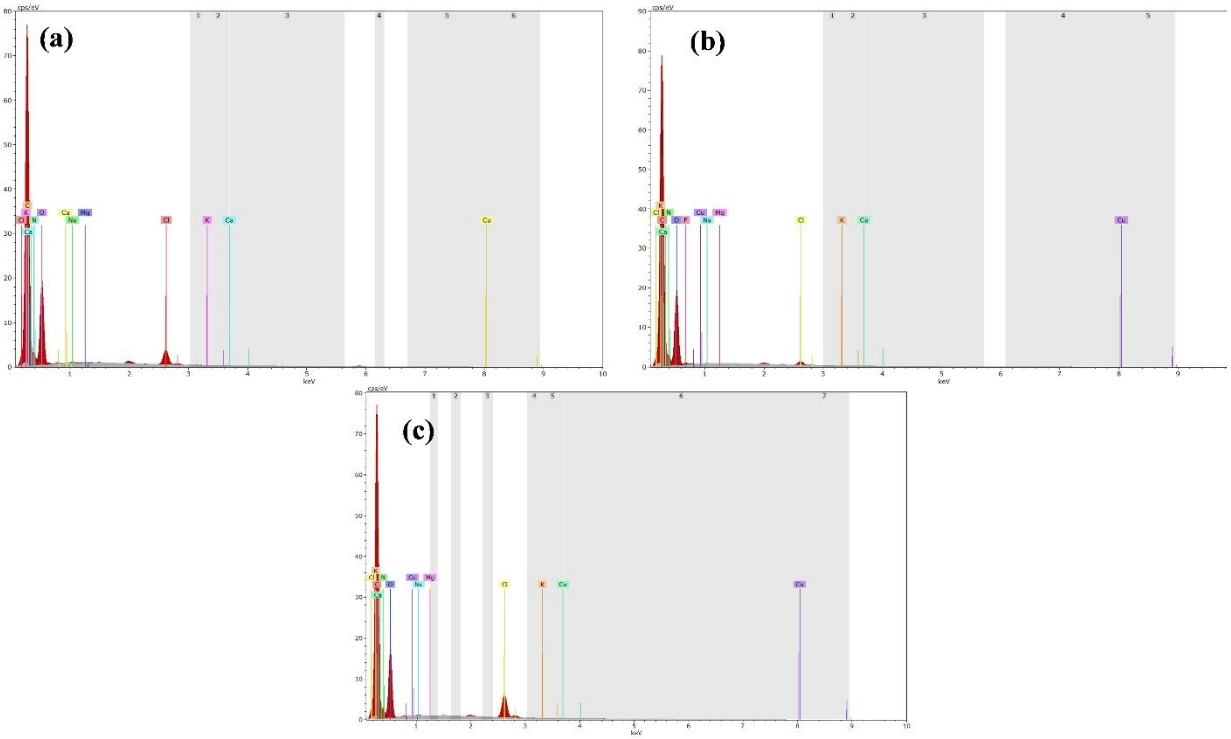

Figure S7. EDX analysis of sawdust@MnO_2@PmPD, (a) BA, (b) AA, and (c) Remodification.

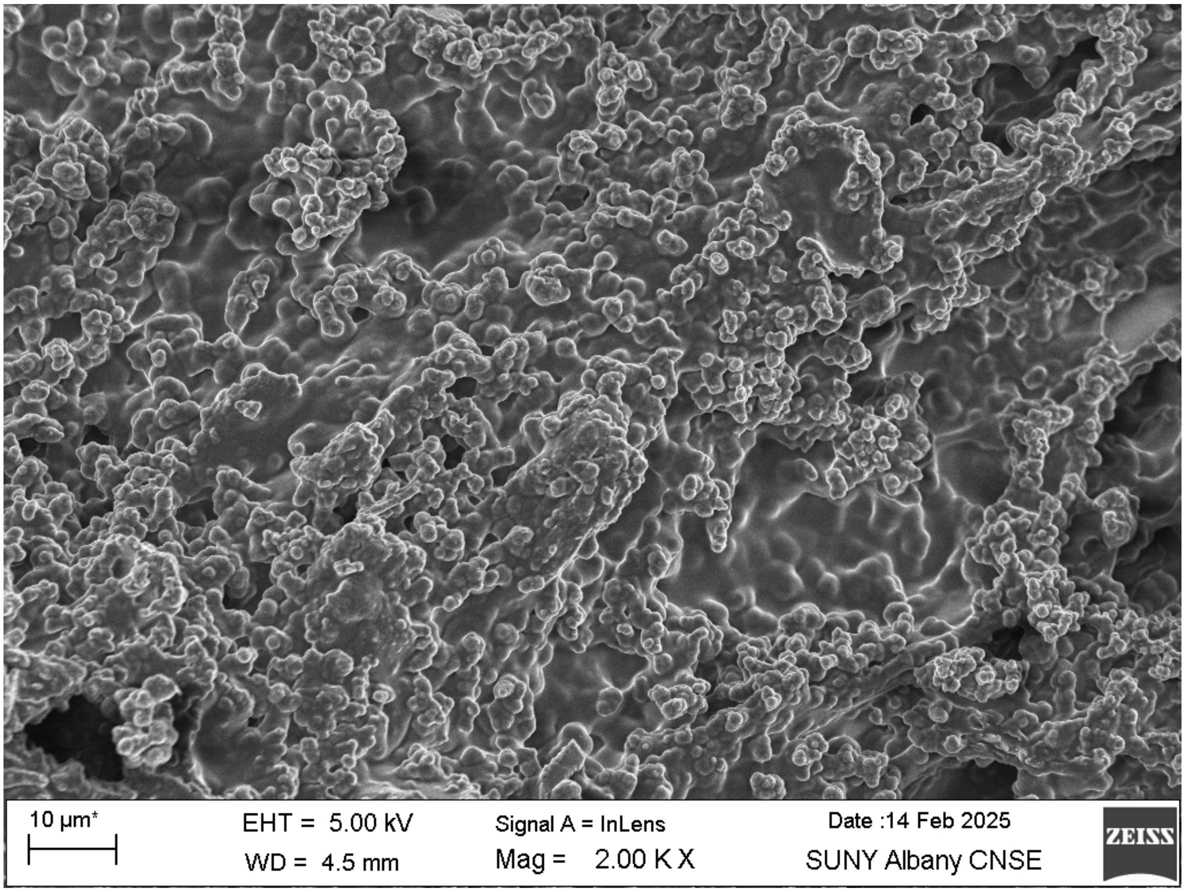

Figure S8. Remodification of the adsorbent

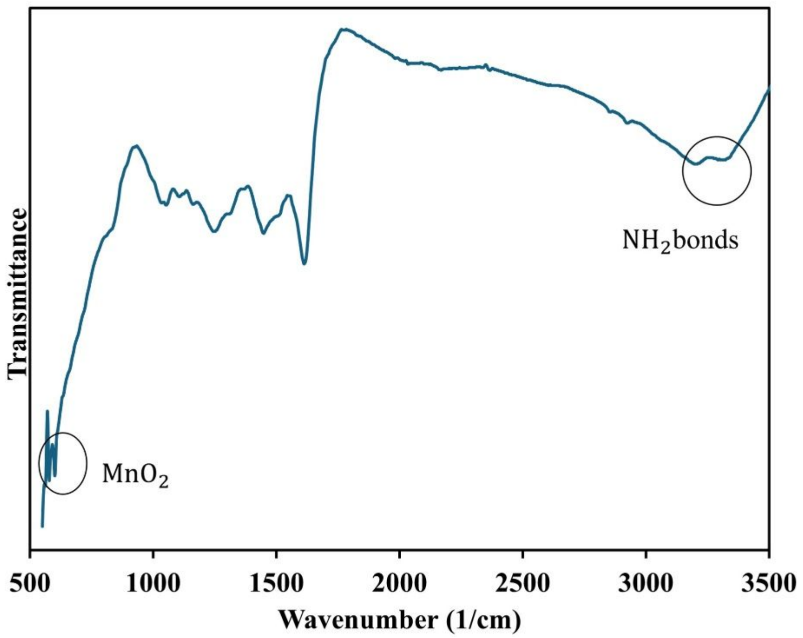

Figure S9. The FT-IR examination of the remodified adsorbent using the spent mPD solution.

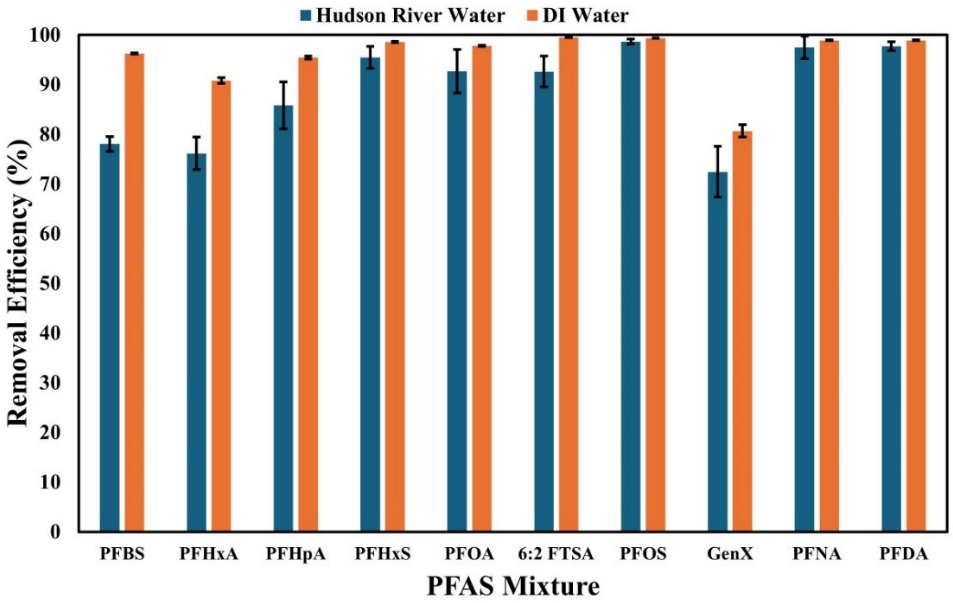

Fig. S10. Application of the sawdust/MnO_2/PmPD on removing PFAS in river water.

References:

[1] A. K. Ilango *et al.*, "Enhanced removal of PFAS in water using activated ZIF-8 carbons: High adsorption efficiency, repeatable regenerability and reusability," *Chem. Eng. J.*, vol. 507, p. 160192, Mar. 2025, doi: 10.1016/J.CEJ.2025.160192.

[2] E. R. Christensen, Y. Wang, J. Huo, and A. Li, "Properties and fate and transport of persistent and mobile polar organic water pollutants: A review," *J. Environ. Chem. Eng.*, vol. 10, no. 2, p. 107201, Apr. 2022, doi: 10.1016/J.JECE.2022.107201.

[3] E. Steinle-Darling and M. Reinhard, "Nanofiltration for trace organic contaminant removal: Structure, solution, and membrane fouling effects on the rejection of perfluorochemicals," *Environ. Sci. Technol.*, vol. 42, no. 14, pp. 5292–5297, Jul. 2008,

doi: 10.1021/ES703207S/SUPPL_FILE/ES703207S-FILE003.PDF.

[4] "(PDF) Environmental Risk Evaluation Report: Perflurooctanesulphonate (PFOS)." Accessed: May 22, 2025. [Online]. Available: https://www.researchgate.net/publication/268256525_Environmental_Risk_Evaluation_Report_Perflurooctanesulphonate_PFOS

[5] S. Kim *et al.*, "PubChem in 2021: new data content and improved web interfaces," *Nucleic Acids Res.*, vol. 49, no. D1, pp. D1388–D1395, Jan. 2021, doi: 10.1093/NAR/GKAA971.

[6] P. S. Pauletto and T. J. Bandosz, "Activated carbon versus metal-organic frameworks: A review of their PFAS adsorption performance," *J. Hazard. Mater.*, vol. 425, p. 127810, Mar. 2022, doi: 10.1016/J.JHAZMAT.2021.127810.
